# Improved Self-Cleaning Properties of Photocatalytic Gypsum Plaster Enriched with Glass Fiber

**DOI:** 10.3390/ma12030357

**Published:** 2019-01-24

**Authors:** Kamila Zając, Magdalena Janus, Antoni W. Morawski

**Affiliations:** 1 Faculty of Civil Engineering and Architecture, West Pomeranian University of Technology, 70-311 Szczecin, Poland; kamila.zajac@zut.edu.pl; 2 Faculty of Chemical Technology and Engineering, West Pomeranian University of Technology, 70-310 Szczecin, Poland; amor@zut.edu.pl

**Keywords:** self-cleaning, gypsum plaster, glass fiber, photocatalysis

## Abstract

In the study the self-cleaning properties of photoactive gypsum plasters are presented. The modified gypsum plasters were obtained by addition of 1 and 3 wt.% of nitrogen-modified titanium dioxide (TiO_2_/N) and 0.1, 0.3, and 0.5 wt.% of glass fiber. The self-cleaning ability of the obtained materials was tested during two dyes decomposition: Methylene Blue (MB) and Reactive Orange (RO). It was found that presence of glass fiber increased photocatalytic activity of modified gypsum plasters, which may be due to the fact glass fiber may act as ducts for light and transport it to sites screened by TiO_2_ or glass fiber can retard charge recombination. Moreover, unexpectedly the addition of glass fiber did not increase the mechanical properties of modified gypsum plasters, which may be because gypsum does not strongly adhere to the surface of glass fibers.

## 1. Introduction

**Figure 1:**
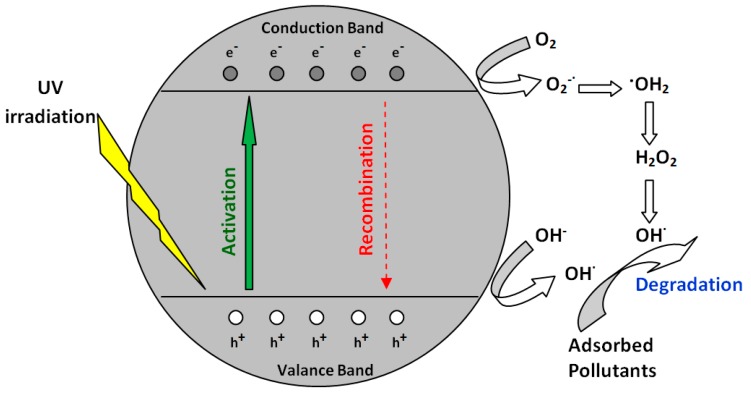
Scheme of the photocatalysis.

Recently, the heterogeneous photocatalysis using TiO_2_ became a promising technique for the removal of major pollutants [1,2]. The photocatalytic process is trigged by appropriate irradiation (ultraviolet for unmodified TiO_2_) which leads to the generation of electron–hole pairs. Produced positive holes and free electrons may subsequently either recombine or form high reactive species (e.g., •OH) which initiate degradation of adsorbed pollutants [3]. The scheme of the photocatalytic mechanism is illustrated in Figure 1.

Moreover, significant efforts have been made to improve the photocatalytic response. One promising way is the modification of TiO_2_ by doping with various elements such as non-metals (N, C, S) or metal ions (Fe, Mn, Cr, Ni) [4]. For example, a higher photocatalytic activity of carbon-doped TiO_2_ resulted from the adsorption enrichment of pollutants and the effective charge separation due to carbon electronic conduction capability [5]. Apart from TiO_2_, other semiconducting metal oxides and metal sulfides also show their photocatalytic performance because of their unique bandgap feature. The application of novel synthetic methods can lead to obtaining mesoporous metal sulfides with enlarged surface area, enhanced mass transfer, and more exposed active sites resulting in improved photocatalytic performance. In addition, some works indicated the increase of photocatalytic activity by the deposition of other semiconductors onto TiO_2_ surface [6]. The intensive development of novel materials, such as metal–organic frameworks, also indicates their photocatalytic action towards pollutant degradation [7]. The broad range of research shows the high demand for the new photocatalytic materials with an enhanced photocatalytic response.

The use of photocatalysts, together with building materials, started in the early 1990s and over the last years has attracted many industrial branches [8]. The data indicates that the sales of photocatalytic building materials accounted for 60% of the photocatalytic market share in Japan (2003) [9]. The analyses carried out in 2016 indicated that the global photocatalyst market is expected to reach USD 4.58 billion by 2025 and the self-cleaning application is going to dominate the photocatalytic branch [10]. Photocatalytic additives have been used in exterior construction materials, such as cement mortar and concrete, as well as in tiles or paving blocks, that involve their self-cleaning properties and air purification [11].

The fact that humans spend approximately 90% of the time indoors indicates the importance of the development of photocatalytic building material dedicated to interior applications, such as gypsum. Gypsum plaster is extensively used to make panels and coatings because of its numerous advantages, such as lightweight construction, thermal and sound insulation, fire resistant, and the creation of a healthy microclimate inside buildings [12,13]. However, gypsum plaster is relatively weak in tension showing brittle behavior [14].

To optimize the mechanical and physical properties of gypsum products, the reinforcement with various types of fiber has been proposed [12]. These fibers can be classified into two groups as natural (carbon, jute, cotton fiber, etc.) and manufactured origins (glass, polypropylene fiber) [13,14,15]. Among them, the most important material used in composites is glass fiber [16]. The commercially available Glass Fiber Reinforced Gypsum, which is a composite from naturally occurring mineral gypsum combined with chopped glass fibers, deserves attention [17,18]. The effect of the glass content on the properties of gypsum composites and its durability has been studied by some authors [19]. Glass fiber is randomly dispersed in the plaster matrix or applied in the form of fabrics. Saliamian et al. [16] investigated the effect of the directions of warp and weft of woven E-glass fabric on mechanical properties of gypsum composite. They concluded that samples in which the fabrics were applied showed increased resistance to bending and tensile forces compared with the plain samples. Moreover, another report [14] showed that the gypsum plaster with glass fiber showed better water-resistance than the unreinforced plaster when immersed in water. 

In photocatalytic tests, organic dyes are often used as model pollutants because of their complex structure and simplicity in monitoring their degradation rate [20]. Especially, azo dyes show their dominance in global production (50–70% of the textile market) [21]. However, methylene blue is utilized by Japanese industrial standards for evaluation of self-cleaning surfaces (JIS-R-1703-2:2007) [22]. In this work, the azo and thiazo dyes were applied as model pollutants.

The major objective of this research was to develop gypsum photocatalytic materials dedicated to interior applications. To obtain the materials the combining of gypsum plaster, modified TiO_2_/N photocatalyst, and glass fiber was considered. In the present paper, the results of photocatalytic tests are presented including various doses of photocatalyst and glass fiber as well as different model pollutants and two types of irradiation. Especially, the results concerning the presence of glass fiber and its influence on not only mechanical but also photocatalytic properties of the obtained gypsum materials are discussed.

## 2. Materials and Methods 

Conventional commercially available gypsum plaster (Knauf MP 75, Iphofen, Germany) was used as a matrix substrate. The modifying agents of the gypsum substrate were the pre-formed TiO_2_/N photocatalyst and/or glass fiber. It was used the E-glass type (Rozenblat company, Mosian, Poland), which is alumino-silicate glass, chopped with a length of 12 mm. As model organic pollutants to photocatalytic tests Methylene Blue (MB) and Reactive Orange 20 (RO) were used supplied by Boruta-Kolor Sp. z o.o., Poland (detailed dyes characteristic in Table 1.).

The synthesis of TiO_2_/N photocatalyst was performed from the commercial amorphous titania supplied by Grupa Azoty Zakłady Chemiczne “Police” S.A., Tarnów, Poland. The detailed procedure is described elsewhere [23]. In short, the starting titania was treated with diluted ammonia water (2.5 wt.%) as a nitrogen source at 100 °C and autogenous pressure using the HEL Ltd. Autolab E 746 installation (HEL Ltd., Borehamwood, UK). The obtained suspension was rinsed with distilled water, filtered, and the obtained slurry was heated for 24 h at 100 °C. Then the photocatalyst was ground using an agate mortar. Additionally, the commercial Aeroxide TiO_2_ P25 (Evonik Industries, Essen, Germany) was used as a reference photocatalyst.

Gypsum plates to administer the photocatalytic tests were prepared according to the following procedure:

(1) The mechanical mixing the pure gypsum plaster with different amounts of TiO_2_/N photocatalyst (1 wt.% or 3 wt.%) or the reference P25 photocatalyst (1 wt.%);
(2) The blending of the homogenous powders with distilled water in an appropriate water/binder ratio;
(3) The addition of glass fiber to the pastes of the selected samples (from 0.1 wt.% to 0.5 wt.%);
(4) The pouring of the obtained pastes into silicone moulds (20 mm × 20 mm × 6 mm);
(5) The demolding of plates after 1 day of drying at room temperature;
(6) The drying of plates at 40 °C to dry matter.

The photocatalytic samples were assigned as P samples, where the first number is associated with the TiO_2_/N loading (e.g., 1 wt.%) and the second number is associated with the glass fiber loading (e.g., 0.1 wt.%), what gives P-1-0.1. It is worth pointing out that as reference samples were analyzed the unmodified gypsum plaster (UM), the gypsum plaster with commercial P25 photocatalyst (CM-P25) and the gypsum plaster with glass fiber without any photocatalysts (UM-0.1, UM-0.3, UM-0.5).

Apart from the photocatalytic activity of prepared materials, their mechanical properties and microscopic structure were also studied. Flexure and compressive strength measurements were conducted using Walter + bai, Combined Compression and Bending Testing Machine Type OB 3000/200. Whereas the elemental analysis of the chosen specimens was determined with cold field emission scanning electron microscope (CFE-SEM Hitachi SU8020, Schaumburg, IL, USA) equipped with energy dispersive X-ray analyser (EDX, Thermo Fisher Scientific, Inc., Hampton, NH, USA). Before the analyses, the samples were excited with electrons of 30 kV energy.

The photocatalytic activity of the obtained plates was evaluated by monitoring the degradation process of two dyes different in their chemical structure and color. The gypsum samples were dipped into 4 cm^3^ volume of the selected dye aqueous solution (MB or RO at a concentration of 100 mg/dm^3^) for 0.5 h. Successively, the imbued plates were dried at 40 °C for 24 h. Photocatalytic experiments were performed in several replicates (between 2 and 5).

The photocatalytic tests were carried out under two sources of irradiation (UV or visible). The distance between samples and irradiation sources was 25 cm. The first series of stained gypsum plasters were treated with UV irradiation (6 × 15 W, Cleo, Berlin, Germany) with the cumulative radiation intensity of 83.4 W/m^2^ UV and 100 W/m^2^ Vis for the duration of 40 h. In the next series of experiments the visible source was applied (two halogen bulbs INQ, Brzesko, Poland, 70 W each) with the cumulative radiation intensity of 0.1 W/m^2^ and 176.4 W/m^2^ for UV and Vis, respectively, during the longer time up to 400 h.

The degradation rate was determined by the colorimetric method using colorimeter CP-21 (tri-color). In the method, the color changes have been expressed in the CIELAB system (Comission Internationale de l’Eclairage). The CIELAB color space is defined by three different chromatic coordinates: L*, a*, and b*, where L* indicates lightness in range 0 to 100 from black to diffuse white; a* indicates negative values towards green and positive values towards red; b* indicates negative values towards blue and positive values towards yellow [24]. Colorimetric calculations were carried out on the basis of procedure adapted from the literature [25]. At the beginning of all photocatalytic experiments the color variation between the original specimens and the treated ones by dye solution have been calculated using the following equation:

(1) $$\Delta E=\sqrt{{\left(L_{0}^{*}-L^{*}\right)}^{2}+{\left(a_{0}^{*}-a^{*}\right)}^{2}+{\left(b_{0}^{*}-b^{*}\right)}^{2}}$$

In the Equation (1) *L*_0_*, *a*_0_*, and *b*_0_* and L*, a*, b* are color coordinates of original specimens and treated ones by dye solution, respectively. After irradiation, the same formula was applied but in this case L*, a*, b* were substituted by *L*_t_*, *a*_t_*, *b*_t_*, which are color coordinates of specimens treated by dye solution and irradiated during the defined period. The photocatalytic efficiency of dye degradation was measured during time using the following equation:

(2) $$R_{E}=\frac{|\Delta E_{t}^{*}-\Delta E_{0}^{*}|}{\Delta E_{0}^{*}}\times 100$$

In the Equation (2), *R_E_* is the photocatalytic efficiency expressed as percentage, Δ*E_t_* is the average variation between the measured color after the defined period of irradiation time and the original surface color before dye application, and Δ*E*_0_ is the average color variation between the measured color after dye application and the original surface color.

## 3. Results and Discussion

### 3.1. Colour Variation of Plates before Photocatalytic Processes

At the beginning of colorimetric measurements, the changes between initial plates were determined. It appeared that the dose of 1 wt.% photocatalyst in plaster matrix did not influence the color of the initial plates. However, after application of TiO_2_/N in the amount of 3 wt.% in gypsum plaster the plates become a little whiter (higher L*) and less red and blue (lower a* and b*) with total color variation equal to Δ*E* = 2.12, slightly noticeable by naked eye. Presence of glass fiber in gypsum plaster did not influence on the surface color of plates.

Staining of gypsum plates with model pollutants involved the significant increase of Δ*E*, which can be observed in Table 2. Especially, the MB dye changed the surface color of initial plates achieving about Δ*E* = 32. After staining, the color variation of specimens’ surfaces was a little higher in the case of unmodified plaster than for the treated ones with photocatalyst and/or glass fiber. It indicated that the modification of gypsum plaster slightly limited the pollutions’ adsorption on the surface of the building material, which initially favors the maintenance of purity. It ought to be stressed that the standard deviations never exceeded 3%. Therefore, in the following results, only mean values are presented. 

The next observation refers to bleaching of dyes on the surface of unmodified plates under UV irradiation. The decrease of Δ*E* value, corresponding to natural reduction after irradiation exposure, was noticeable. For UM samples after 40 h of UV irradiation Δ*E* reached 29.26 (Δ*E* = 32.02 after staining) and 19.67 (Δ*E* = 25.78 after staining) for MB and RO, respectively. Visible light showed a lower bleaching effect. The similar observations of untreated limestones have also been made by other authors [26]. Meanwhile, Graziani et al. [27] described a light discoloration of MB on untreated stained fired clay bricks. It was detectable because some of the dyes are sensible to irradiation and tend to degrade by themselves. In their review work, Munafo et al. [24] indicated that in most cases, the applied dyes on untreated architectural stones were partly decomposed by simple exposure to UV irradiation. 

### 3.2. Photocatalytic Properties of Gypsum Plates under UV Irradiation

#### 3.2.1. Effect of Photocatalyst Type and Model Dye Structure

**Figure 2:**
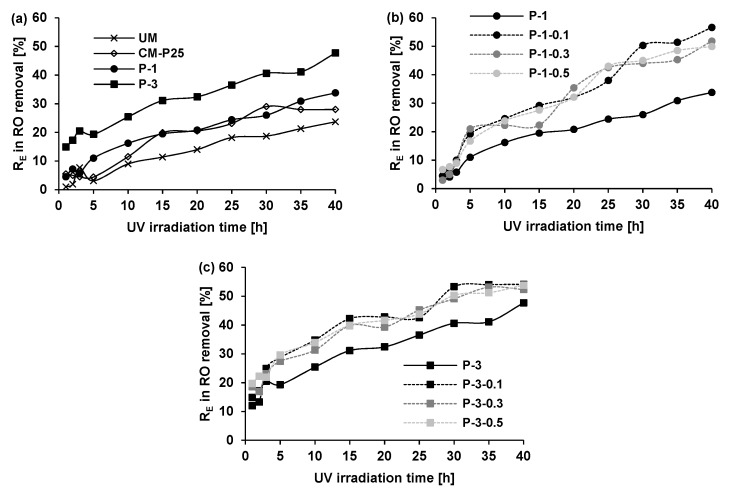
Photocatalytic degradation of Reactive Orange (RO) dye from gypsum surface as a function of UV irradiation time. (**a**) UM-unmodified gypsum plaster; CM-P25 gypsum plaster with 1wt%TiO_2_-P25; P-1, P-3 gypsum plaster with 1 and 3wt.% of TiO_2_/N; (**b**) P-1 gypsum plaster with 1 wt.% of TiO_2_/N; P-1-0.1, P-1-0.3, P-1-0.5 gypsum plaster with 1 wt.% of TiO_2_/N and 0.1, 0.3, 0.5 wt.% of glass fiber, respectively; (**c**) P-3 gypsum plaster with 3 wt.% of TiO_2_/N; P-3-0.1, P-3-0.3, P-3-0.5 gypsum plaster with 3 wt.% of TiO_2_/N and 0.1, 0.3, 0.5 wt.% of glass fiber, respectively.

**Figure 3:**
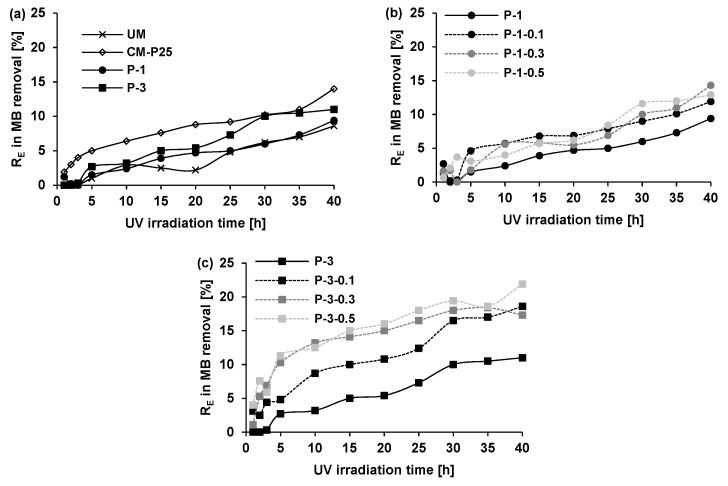
Photocatalytic degradation of Methylene Blue (MB) dye from gypsum surface as a function of UV irradiation time. (**a**) UM-unmodified gypsum plaster; CM-P25 gypsum plaster with 1wt%TiO_2_-P25; P-1, P-3 gypsum plaster with 1 and 3wt.% of TiO_2_/N; (**b**) P-1 gypsum plaster with 1 wt.% of TiO_2_/N; P-1-0.1, P-1-0.3, P-1-0.5 gypsum plaster with 1 wt.% of TiO_2_/N and 0.1, 0.3, 0.5 wt.% of glass fiber, respectively; (**c**) P-3 gypsum plaster with 3 wt.% of TiO_2_/N; P-3-0.1, P-3-0.3, P-3-0.5 gypsum plaster with 3 wt.% of TiO_2_/N and 0.1, 0.3, 0.5 wt.% of glass fiber, respectively.

Figure 2 and Figure 3 show the self-cleaning efficiency *R*_E_ of tested specimens during UV irradiation. Moreover, the RO and MB degradation on the samples can be visible by the naked eye in Table 3. In most cases, the photocatalytic activity of plates containing titanium dioxide is well evident in comparison to the unmodified gypsum plaster results (UM). However, the application of TiO_2_/N in dose of 1 wt.% in gypsum plaster (P-1 in Figure 3a) gave only a slightly better effect than the simple bleaching of MB. 

It is worth pointing out that the highest differences in degradation rate from the kinetic point of view were observed during the first 5 h of irradiation. After 5 h the degradation speeds were comparable for the majority of samples. However, the final photocatalytic effectiveness was various for different systems. At the beginning of RO photocatalytic removal, the P-1 seemed to degrade this dye to a higher degree than using the CM-P25 with commercial photocatalyst (Figure 2a). Prolongation of the process over the period of 15 h caused comparable effectiveness between the two photocatalytic materials. Photocatalytic tests using MB (Figure 3a) showed clearly better results for CM-P25 than even for P-3 with a dose of 3 wt.% of modified photocatalyst.

The high activity of CM-P25 during MB photocatalytic degradation can be associated with different physicochemical properties between P25 and TiO_2_/N, such as different isoelectric point, which amounted to 6.7 for TiO_2_/N and 5.9 for P25 [28] and, consequently, different interaction with dyes molecules. Moreover, in our previous report [29] it was proved that at the beginning of irradiation more •OH radicals are formed on the surface of P25 than on TiO_2_/N. However, as the duration of irradiation increases, a higher linear increase of •OH radicals was noted for TiO_2_/N sample. •OH radicals, as the main oxidative species in photocatalysis, were measured using the indirect fluorescence method. Considering the differences in the dyes photocatalytic degradation, the surface area and porosity of the photocatalytic materials should be analyzed because the photocatalysis is the surface reaction and the adsorption of the pollutants on the surface is the initial stage of the photocatalytic reaction. In our previous report [30], it has been shown that P25 and TiO_2_/N differ significantly in surface area. A nearly five times higher S_BET_ surface area was observed for TiO_2_/N (259 m^2^/g) than for P25 (55 m^2^/g). Additionally, in our work, it has been shown that the loading of both photocatalysts up to 5 wt.% to gypsum materials did not influence the final surface area and porosity of the modified gypsum materials. The increase in surface area and porosity have been observed using doses as 10 and 20 wt.% of the photocatalysts to gypsum matrix. It appeared that the reasons for the promoted photocatalytic capability of CM-P25 during MB degradation were the variations in surface acid-base properties and rate of •OH generation on photocatalysts surfaces during irradiation. 

Generally, in the literature [31,32], MB is more susceptible to photocatalytic degradation than azo dyes. In the work of Nguyen et al. [32], the prepared palladium–doped titanium dioxide catalyst always degraded MB faster than azo dye, Methylene Orange. It was explained by the stability of the by-products created during azo dye degradation because azo bond (–N=N–) was hardly cleaved. Meanwhile, in our work the application of TiO_2_/N gave the opposite dependences, indicating the much easier and faster disintegration of azo bond in RO in comparison to the structure of MB. After 10 h of UV irradiation, the P-1 sample showed insignificant MB degradation (2.4%) compared to RO dye (16.2%).

Notably, after 40 h the degradation rate of RO was over 3.5 times higher than MB (33.8% for RO vs. 9.4% for MB). The important structural difference between the molecules of the two dyes is that in the case of RO, there is an azo group (–N=N–), which is not present in the MB molecule. Meanwhile, in MB a thiazine structure (organic ring of four carbons, one nitrogen and one sulfur atom) is present in the absence of the azo bond [31]. It has been also reported [32] that it is difficult for any single photocatalyst to degrade both anionic and cationic dye pollutants with equal ease because they show completely different surface properties under identical conditions.

It should be stressed that in the first period of irradiation the coloration of plates by MB was even higher than after staining directly. It indicates the soaking the dye solutions into the plaster structure and moving of dye molecules to the surface of plates during irradiation. This may be the next possible reason for differences in both dyes degradation effectiveness. 

#### 3.2.2. Effect of Photocatalyst Type and Model Dye Structure

Considering then the effect of TiO_2_/N loading on the photocatalytic performance of the gypsum materials, it was observed that increasing the TiO_2_/N content from 1 wt.% to 3 wt.% enhanced the photocatalytic activity, as expected. However, the influence was also much stronger in relation to RO than MB. Even when increasing TiO_2_/N content in gypsum plaster from 1 wt.% to 3 wt.%, the increase of MB degradation was as low as 1.6% after 40 h of UV irradiation, whereas in the case of RO the same elevation of photocatalyst dose resulted in nearly 14% higher RO removal from sample surfaces after the same time of irradiation. According to literature reports [33,34], it is worth mentioning that the excess of the photocatalyst can limit photocatalytic effectiveness by blocking the light accessibility to photocatalyst particles as well as leading to the increase of charge recombination rate. In most cases, there is an optimal amount of photocatalyst for the determined photocatalytic system [35] and the consideration of higher photocatalyst dose is unfounded. Thus, in the work, the photocatalyst loading in gypsum plaster did not exceed 3 wt.%.

#### 3.2.3. Effect of Glass Fiber Presence

The initial intention of glass fiber incorporation into gypsum plaster structure was the enhancement of mechanical properties of the obtained photocatalytic samples. However, the addition component in gypsum building material can have an influence on the photocatalytic properties. 

The influence of glass fiber presence on photocatalytic gypsum plaster are presented in Figure 2b,c and Figure 3b,c. All this date indicates that the addition of glass fiber to photocatalytic gypsum plates improves their photocatalytic effectiveness even up to two times. The influence of glass fiber can be discussed based on type of dye and dose of photocatalyst in gypsum plaster. The dose of glass fiber did not have a substantial influence on the results. Glass fiber enhanced RO degradation using as little as 1 wt.% of TiO_2_/N in gypsum plaster. In this case, the RO was over half decomposed (50.3% of RO removal) within 30 h of UV, which significantly exceeded the analogous results without the use of glass fiber (26.0% of RO removal). During the application of higher TiO_2_/N loadings in the samples, the influence of glass fiber was also clearly not so spectacular. Here it is important to stress that after 40 h of UV irradiation the results of RO removal was comparable (~55%) regardless of photocatalyst loading as long as glass fiber was present in building material structure. It was found that glass fiber allowed the photocatalyst dose in gypsum plaster to be limited. Using 1 wt.% of TiO_2_/N and glass fiber in gypsum plaster provided better efficiency of RO decontamination (56.6%) than using 3 wt.% of TiO_2_/N in samples without glass fiber (47.7%). As discussed in Section 3.2.1. MB is a pollutant with a different structure and properties than RO, which involved other observation of MB changes with reference to gypsum plates with glass fiber. Admittedly the presence of glass fiber generally also improved photocatalytic results but to a greater extent for plates with 3 wt.% of TiO_2_/N (improvement of about 9%). For MB dye the photocatalyst dose of 1 wt.% in gypsum plaster appeared to be insufficient even after glass fiber application (improvement only about 3%). 

It should be stressed that the parallel studies using unmodified samples with various glass fiber loading were carried out (UM-0.1, UM-0.3, UM-0.5). It appeared that regardless of glass fiber dose in pure gypsum plaster the dyes degradation was comparable to results for reference UM. 

The main component of the applied glass fiber is SiO_2_. Thus, the interaction of TiO_2_ and SiO_2_ should be taken into account. The unique synergistic effect of SiO_2_–TiO_2_ nanocomposite structure was observed by many authors [36,37]. The improved photocatalytic activity of TiO_2_ by addition of SiO_2_ was explained in different ways:

(1) As the increase of available surface of photocatalyst increased it allowed an increase in adsorption of pollutants molecules [38];
(2) As the increase of amount of surface adsorbed water and hydroxyl groups, which are necessary in photocatalytic processes [39];
(3) As the increase of dispersion of TiO_2_ particles [40].

However, SiO_2_ was mainly used as a support for TiO_2_ particles. TiO_2_ particles were often immobilized on the surface of SiO_2_ through a layer self-assembly procedure [41]. Another example is the synthesis of silica nanospheres decorated with TiO_2_ nanocrystals by a hydrothermal method [36]. The monodispersed teardrop-shaped core-shell SiO_2_/TiO_2_ nanoparticles were also fabricated via the sol–gel method [42]. However, in our case, the glass fiber was the additional component of building material without the intended interaction with the photocatalyst. It is also possible that in our study the enhanced degradation rate of pollutants on the plates with glass fiber can be associated with the increased delocalization of charge carriers. It is known from the literature that electron–hole charge separation plays a key role in achieving high quantum photocatalytic efficiency [43]. A number of researches have presented that the combination of SiO_2_ and TiO_2_ is efficient to retard charge recombination [44,45,46]. Namely, glass fiber in photocatalytic gypsum plaster can enhance charge separation, limiting the electron–hole recombination. Thereby, electrons and holes can degrade the organic compounds directly or indirectly by triggering a series of reactions leading to the formation of oxidative species, e.g., •OH [47]. 

However, the adjunctive effect might have an influence on the enhanced photocatalytic activity of gypsum plates with glass fiber. Namely, the glass fibers may act as ducts for photons and transport them to sites screened by TiO_2_. Grześkowiak et al. [48] proved that quartz (glass) favors accessibility of irradiation to the deeper regions of the TiO_2_. They examined the transmission of the UV-A radiation through quartz wool, TiO_2_ powder, and TiO_2_ supported on the wool quartz. It appeared that the transmission of the light by the TiO_2_ supported on the quartz exceeded that observed for the TiO_2_ powder alone. On the basis of the above considerations, it is clearly seen that the further development of our photocatalytic materials with glass fiber will require a better understanding of the nature of the enhanced photoactivity phenomena and there is a necessity of further detailed studies.

### 3.3. Photocatalytic Properties of Gypsum Plates under Vis Light 

**Figure 4:**
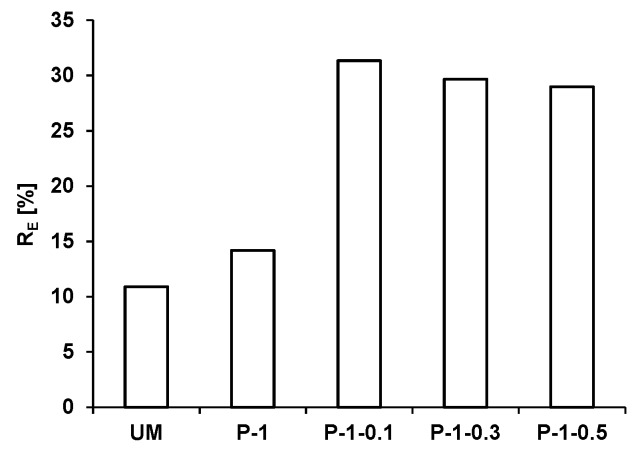
Photocatalytic efficiency during RO removal from gypsum samples under visible irradiation.

Photocatalytic activity under visible light was presented on the representative series of gypsum plates with 1 wt.% of TiO_2_/N during degradation of RO dye (Figure 4). It should be stressed that the application of visible light and colored contaminants involved the additive sensitization effect, which can cause charge excitation and facilitate reactivity [36]. It appeared that the presence of photocatalyst in the gypsum matrix (P-1) scarcely improved the photocatalytic response after 400 h; about 4% (under UV the enhancement achieved even 10% after 40 h). However, surprisingly again the concurrent presence of glass fiber increased RO removal efficiency, which is clearly seen in Figure 4. Using P-1-0.1, the RO degradation rate was over two times higher in comparison to the photocatalytic sample without glass fiber (the increase from about 15% to about 30%). As discussed in the previous section, under UV irradiation the same experiments also gave the two-times enhancement of RO removal but in a higher range (from about 30% to 60%). Simultaneously, analogous as under UV irradiation, the photocatalytic efficiency under visible light of UM-0.1, UM-0.3, UM-0.5 was comparable to UM. The results indicated that under visible light the glass fiber also significantly enhance the photocatalytic activity of gypsum plates modified with the photocatalyst. 

On the basis of the results obtained under visible irradiation, the role of glass fiber dose should be discussed. On the one hand, it seems that glass fiber reduces the recombination of photogenerated electron–hole pairs by the charge trapping which enhances photocatalytic activity. On the other hand, too high an amount of glass fiber slightly reduced the photocatalytic activity (Figure 4). When glass fiber loading exceeded a certain limit, it can act not only as charge capture but also become a recombination center or might cover the active sites. Similar observations were described by other authors in case of too high an amount of modifying agent in photocatalyst structure [49].

### 3.4. Mechanical Properties and Microstructure of Modified Gypsum Plaster 

**Figure 5:**
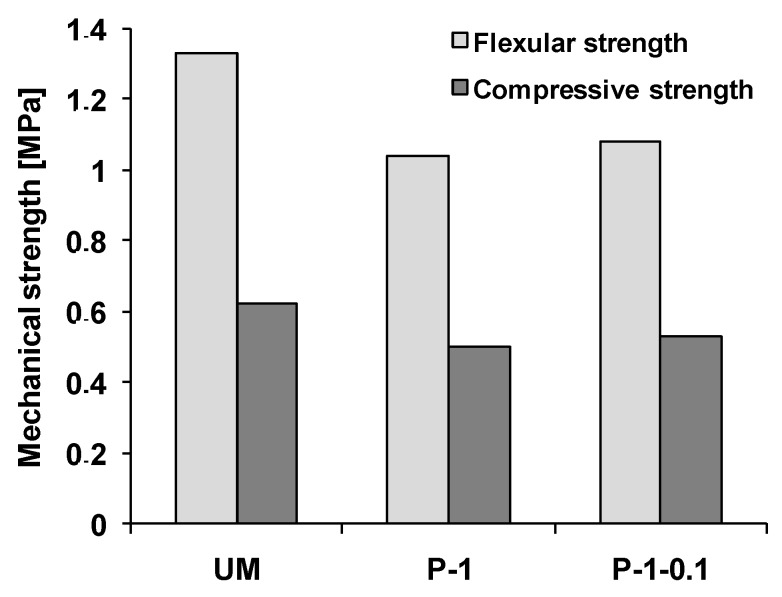
Changes of mechanical properties of gypsum samples after loading of TiO_2_/N and glass fiber.

**Figure 6:**
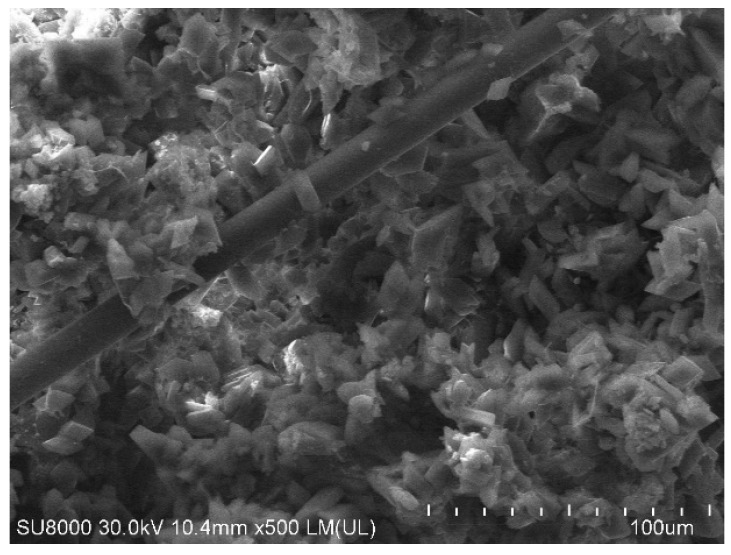
SEM image of gypsum plasters containing 0.5 wt.% of glass fiber.

In Figure 5, the mechanical properties of gypsum samples were presented. It can be clearly seen that the presence of TiO_2_/N in the gypsum matrix adversely decreased both the flexular and compressive strength of about 22% and 19%, respectively. It has to be admitted that the presence of glass fiber was not as effective as expected. The loading of 0.1 wt.% glass fiber in gypsum plaster allowed to increase of flexural and compressive strength scarcely about 4 to 6%. The higher loading of glass fiber in samples also did not produce a significant improvement in mechanical properties. It may be caused by the fact that the glass fiber was not very tightly covered by gypsum plaster. The gypsum plaster with 0.5 wt.% of glass fiber is presented in Figure 6.

## 4. Conclusions

The results proved that the gypsum plasters with TiO_2_/N and additional glass fiber presence showed enhanced photocatalytic performance under UV and visible light irradiation in comparison to the gypsum plasters modified only with the photocatalyst. The glass fiber allowed to obtain an over two times higher degradation rate under both irradiation sources. The photocatalytic removal of two model dyes, Methylene Blue and Reactive Orange, on the prepared gypsum materials corresponded well with the amount of TiO_2_/N particles in range from 1 to 3 wt.%, whereas the amount of glass fiber did not have a substantial influence on the results in range from 0.1 to 0.5 wt.%. The presence of glass fiber may act as ducts for UV light and transport it to sites screened by TiO_2_ or enhance charge separation, which requires further detailed studies. However, the presence of glass did not increase the mechanical properties of gypsum plasters effectively because the components of the gypsum matrix did not cover the glass fiber tightly. 

## Figures and Tables

**Table 1:** Characteristics of dyes.

Dye	Charge in Dissociated Form	Chromophore Group	Chemical Structure
Reactive Orange 20 (RO)	anionic	azo	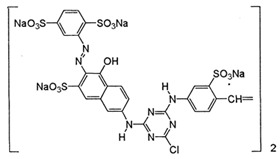
Methylene Blue (MB)	cationic	thiazine	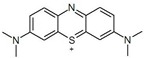

**Table 2:** Aeshtetical properties of exemplary stained surfaces expressed in chromatic coordinates (CIELAB) and color variations (Δ*E*) with standard deviations.

Samples		Initial	After RO	After MB
UM	L*	81.71 ± 0.27	68.05 ± 0.15	62.26 ± 0.33
	a*	1.96 ± 0.07	8.85 ± 0.5	−8.80 ± 0.12
	b*	9.62 ± 0.41	30.36 ± 0.57	−13.67 ± 0.46
	Δ*E*	-	25.78 ± 0.31	32.02 ± 0.53
P-1	L*	80.97 ± 0.93	70.52 ± 1.50	67.47 ± 0.15
	a*	1.88 ± 0.22	8.47 ± 0.28	−7.53 ± 0.13
	b*	9.86 ± 0.27	30.20 ± 0.19	−10.99 ± 0.45
	Δ*E*	-	23.83 ± 0.45	26.53 ± 0.35

**Table 3:** Images of exemplary gypsum plates during the photocatalytic experiments.

Sample	Initial Surface	Stained Surface	After 40 h of UV Irradiation
UM	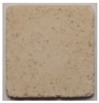	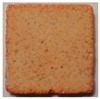	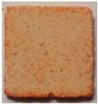
P-1-0.1	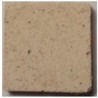	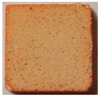	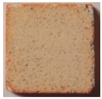
UM	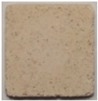	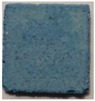	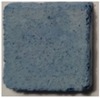
P-3-0.1	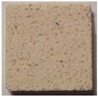	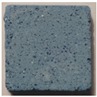	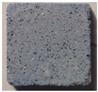
